# The hidden perils of laser lead extractions: Managing internal mammary artery perforation

**DOI:** 10.1016/j.jvscit.2025.101885

**Published:** 2025-06-19

**Authors:** Wingtung M. Ma, Thomas M. Warburton, Alexandra Sim, Alasdair Watson, Rajesh Subbiah, David Evans

**Affiliations:** a Faculty of Medicine, University of New South Wales, Sydney, New South Wales; b Department of Vascular Surgery, St Vincent's Hospital Sydney, Darlinghurst, New South Wales; c Department of Cardiothoracic Surgery, St Vincent's Hospital Sydney, Darlinghurst, New South Wales; d Cardiology Department, St Vincent's Hospital Sydney, Darlinghurst, New South Wales

**Keywords:** Cardiac pacemaker, Complications, Hemorrhage, Iatrogenic AV fistula, Endovascular embolization

## Abstract

Transvenous lead extraction for cardiac implantable electronic devices remains a high-risk procedure among profound evolutions of extraction techniques. Laser-assisted transvenous lead extraction is required with prolonged lead dwell times or failed mechanical extraction. Tertiary subspecialty support is crucial to mediate the small but significant risk of cardiovascular morbidity and mortality. We report a case of laser lead extraction complicated by internal mammary artery perforation and fistulization, salvaged with endovascular intervention. Multidisciplinary subspecialty support, skills, and infrastructure should be used for all these extractions.

With the growing prevalence of cardiovascular diseases, cardiac implantable electronic device implantation has become increasingly common for managing cardiac arrhythmias and heart failure. Chronic cardiac implantable electronic device users are at risk of device complications, which necessitate transvenous lead extractions (TLEs). The most common indications are device infection, lead dysfunction, and symptomatic venous occlusion.^1^

Lead removal can be challenged by tissue fibrosis and calcified binding of leads to the vessel walls and myocardium. Following a staged approach, leads with an extended dwell time (>6 months) or nonextractable by simple mechanical techniques will undergo laser-powered sheath extraction.^2^ Laser sheaths deliver laser energy to the distal end, dissecting adhesions and releasing leads from severe fibrotic encapsulation. The laser technique is safer and more effective than simple traction for selected high-risk patients, notwithstanding some associated complications and mortality risks because of the more complex pathology.^3^^,^^4^ This report presents a case of endovascular salvage of left internal mammary artery (LIMA) perforation and arteriovenous fistula formation between the LIMA and left subclavian vein during laser-assisted lead extraction. Written consent was obtained from the patient to publish deidentified case details and images.

## Case report

**Fig 1:**
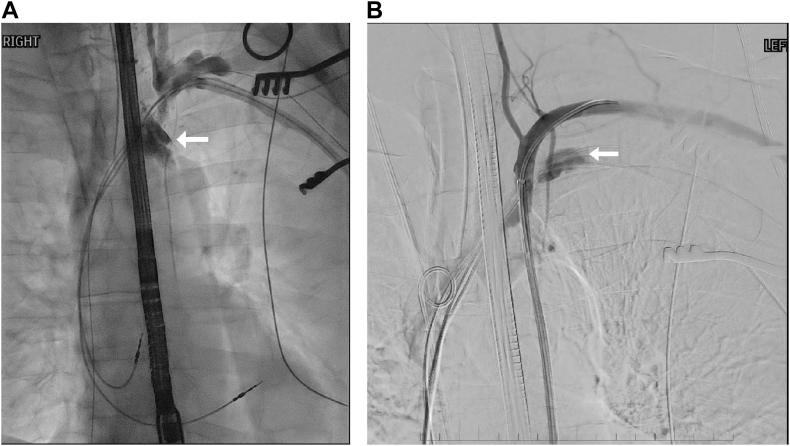
**(A)** Venogram showing superior-mediastinal extravasation from a lacerated proximal left internal mammary artery (LIMA) (*arrow*) and **(B)** selective left subclavian arteriogram showing fistulization between the LIMA and proximal subclavian vein (*arrow*).

A 74-year-old man underwent pacemaker implantation for symptomatic sinus bradycardia and persistent atrial flutter at the age of 64 years with no history of previous coronary artery bypass graft surgery. The original leads (Medtronic 5076, Minneapolis, MN) were inserted via subclavian venous access. At 130 months of indwelling time, right ventricular pacing lead failure and frequent ventricular tachycardia were detected, although the leads were not fractured. He presented to the cardiothoracic surgery unit for an elective TLE using an Excimer laser sheath (Spectranetics, Colorado Springs, CO) to remove the atrial and ventricular leads. A locking stylet was deployed to establish control and stabilization of the leads. A 16F GlideLight laser sheath was operated at 60 mJ, 80 Hz, and advanced at 1 mm/s. Preoperative imaging showed mild stenosis of the left subclavian vein. During lead extraction, we encountered difficulty in advancing the active laser sheath. Pulsatile bright red blood was then noted from the side arm of the sheath. A brief hypotensive episode was controlled with crystalloid fluid and transfusion of 1 U of packed red blood cells. A blood gas sample was obtained from the sidearm of the laser sheath to confirm arterial blood. Intraoperative diagnostic angiography via the side sheath of the laser device confirmed hemorrhage from a perforated proximal LIMA into the superior mediastinum and fistulization with the left proximal subclavian vein (Fig 1). The laser sheath was left in situ, and endovascular salvage of the perforation was commenced through the femoral artery.

**Fig 2:**
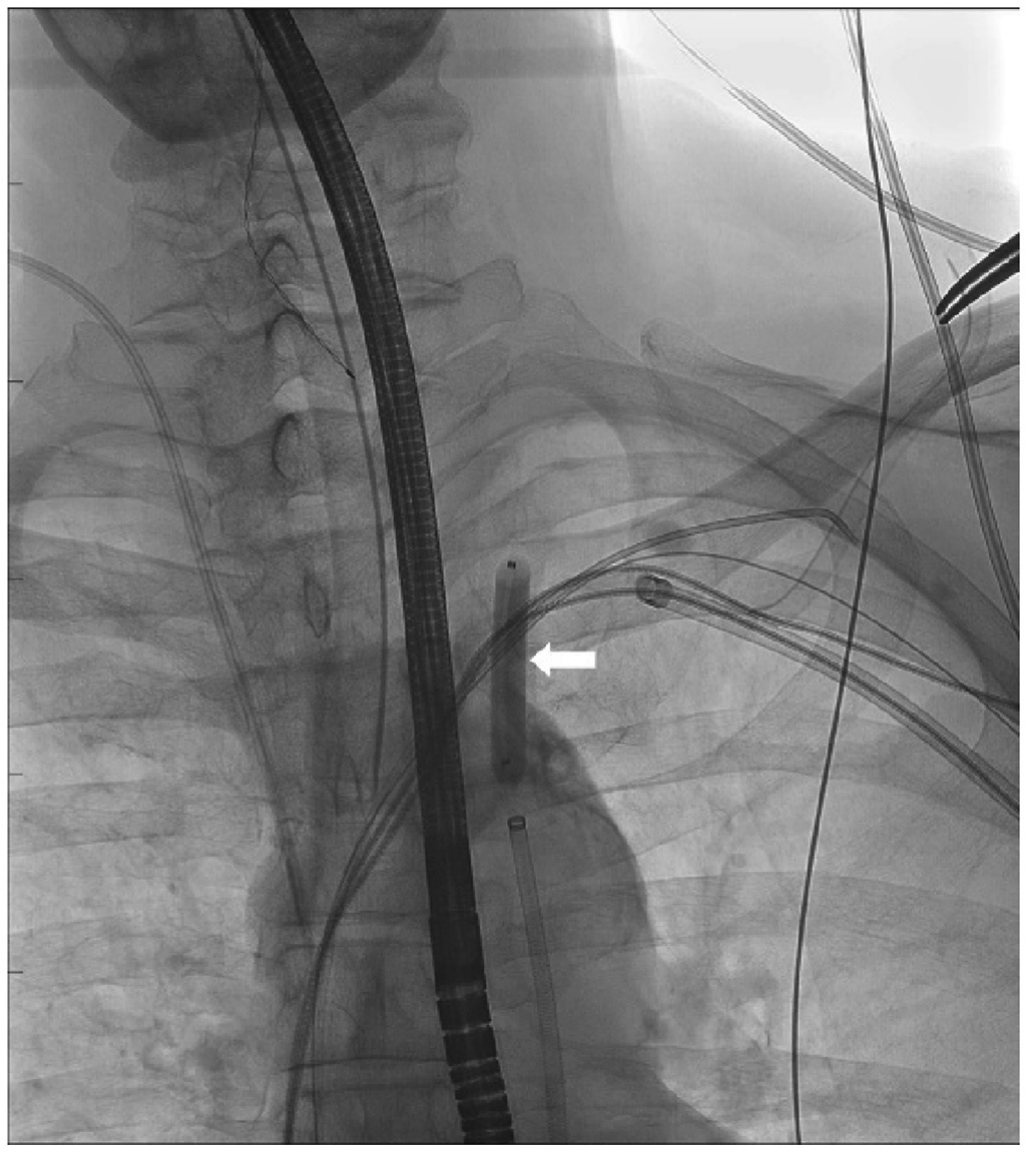
Angiogram showing percutaneous deployment of intravascular balloon (*arrow*) and occlusion of the left subclavian artery to achieve immediate hemostasis.

**Fig 3:**
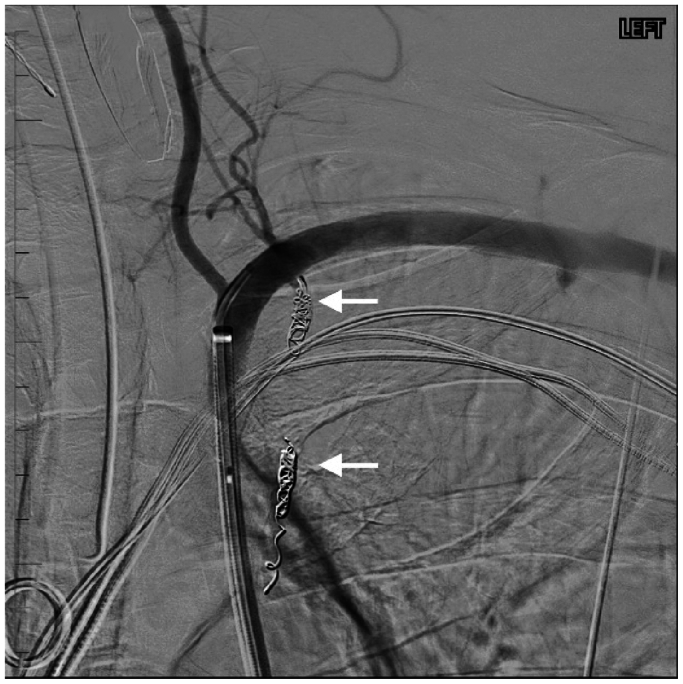
Selective left subclavian arteriogram showing successful coil embolization of the left internal mammary artery (LIMA) with complete closure of the arteriovenous fistula and cessation of hemorrhage. *Arrows* represent coil-embolized inflow and outflow to pseudoaneurysm.

An 8F × 90 cm Destination sheath (Terumo Aortic, Tokyo, Japan) was introduced via the right common femoral artery and positioned in the origin of the left subclavian artery. An 8-mm Abbott Armada balloon (Abbott Vascular, Abbot Park, IL)in the proximal left subclavian artery was used to control the hemorrhage over a 0.35” system (Fig 2). The left IMA was catheterized with a triaxial system including the sheath, a 5F × 125 cm angled Bern catheter (Merit Medical Systems, Jordan, UT) over a 2.6F × 0.018 in × 150 cm angled CXI microcatheter system (Cook Medical, Bloomington, IN). Embolization of the IMA was performed by the trap-door technique, deploying two detachable coils (4 and 5 mm; Interlock, Boston Scientific, Marlborough, MA) from the distal end (back door) of the perforated site to the proximal end (front door). Care is taken during coil deployment to prevent prolapsing through the fistula and causing nontarget embolization to the heart and lungs. Completion angiography confirmed successful control of the hemorrhage and closure of an arteriovenous fistula (Fig 3). A new single-coil implantable cardioverter-defibrillator lead was confirmed to be functional and implanted at the right ventricular apex, abandoning the ventricular pace-sense lead. The old lead was capped, and the new device was implanted in the new subpectoral pocket. The patient was discharged 3 days after lead extraction following a period of inpatient observation. The patient was clinically well at the 1 month outpatient follow-up.

## Discussion

**Fig 4:**
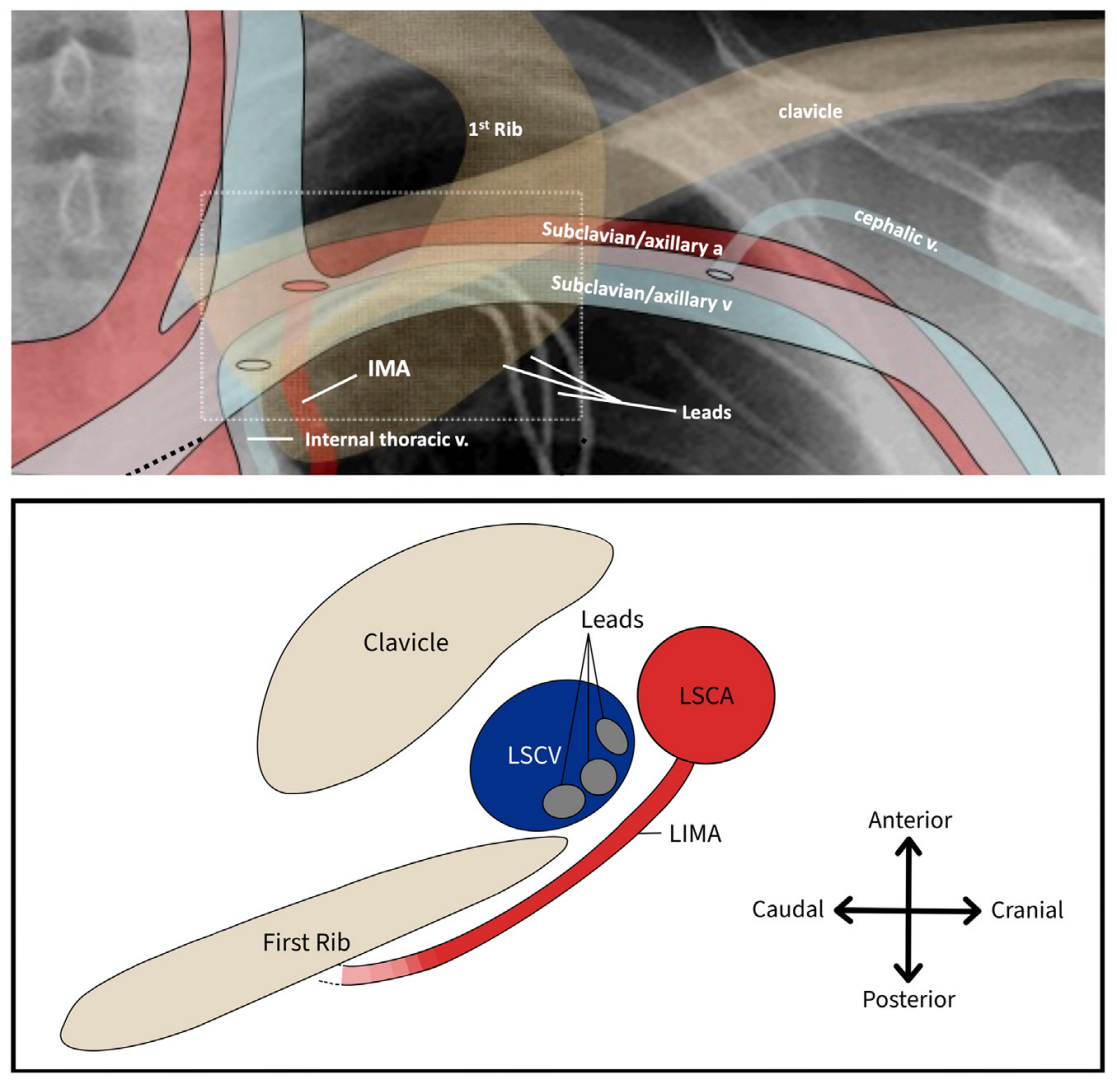
Anatomical relationship between the internal mammary artery and subclavian vein. Adapted with permission from Cruz et al (2011).^5^*IMA*, internal mammary artery; *LCSA*, left subclavian artery; *LIMA*, left internal mammary artery; *LSCV*, left subclavian vein.

The challenges of laser lead extraction pertain to the safe removal of the leads. Mortality related to TLE is approximately 1.5%, with the rate of major complications estimated to be 3.0%.^4^ The binding of leads to the superior vena cava renders the vessel susceptible to tears during the procedure. Although the estimated incidence of superior vena cava tears during TLE is relatively low (0.5%), this entity remains one of the most lethal complications.^3^ Given the risk of major vessel injuries, the laser method is performed selectively among patients with a prolonged lead dwell time, which is associated with higher procedural failure and complication rates with simple mechanical techniques (Fig 4). Vascular injury could be minimized by thorough pre-procedural planning that takes into account preoperative imaging, lead design, dwell time, and laser sheath size. Surgeons may consider performing balloon angioplasty to predilate stenotic lesions using a lower profile laser sheath and following the recommended transit speed (1 mm/s). Readiness to manage complications effectively is imperative. Emergency endovascular interventions and the preparation of a cardiopulmonary bypass machine should be considered in procedural planning to optimize the safety of laser lead extractions.^6^^,^^7^ Alternative surgical approaches to managing acute arterial perfusion include covered stent repair, prolonged balloon inflation, or open surgical repair.

Post-TLE traumatic vascular injuries may present subtly during extraction but can lead to rapid clinical deterioration. Rupture of vessels—in this case, the IMA—can cause instability via blood loss and tamponade or tension. Unlike an arterial stenosis, which maintains the native diameter of the vessel, a fibrotic venous stenosis leads to contraction of the vessel, thereby distorting neighboring structures. In this case, it brought the IMA in close proximity to the Excimer laser sheath as it passed through the stenosed vein. Arterial occlusive balloon techniques are invaluable, as in this case, for stabilizing and resuscitating as a bridge to definitive endovascular treatment from either covered stenting or embolization. To avoid systemic heparinization in this bleeding patient, a wireless plain balloon was inflated with heparinized saline infused through the wire channel of the device into the subclavian outflow, reducing the risks of stroke and thromboembolic events, especially in the vertebral circulation. Balloon occlusion with gentle nominal pressure and size with a compliant balloon is recommended to prevent iatrogenic intima injury from this technique. Selective catheterization of the LIMA was performed using triaxial support (sheath, catheter, and microcatheter), providing superior and safer steerability and pushability from the groin access. Detachable coils are a must for safe deployment in this technique. The seriousness of this injury in the presence of a LIMA graft cannot be understated. In this situation, we extrapolate from the endovascular management of trauma with covered stenting of the perforation to maintain the patency of the graft.

## Conclusions

The demand for laser-assisted TLE is increasing with the growing prevalence of chronic lead implantation. Procedural complications in these selected high-risk patients can be mitigated by preemptive planning of endovascular salvage interventions and a collaborative approach to care.

## Funding

None.

## Disclosures

None.
